# Eugenol and Thymol Derivatives as Antifeedant Agents against Red Palm Weevil, *Rhynchophorus ferrugineus* (Coleoptera: Dryophthoridae) Larvae

**DOI:** 10.3390/insects12060551

**Published:** 2021-06-13

**Authors:** Tay Karh Yan, Asnuzilawati Asari, Siti Aishah Salleh, Wahizatul Afzan Azmi

**Affiliations:** 1 Faculty of Science and Marine Environment, Universiti Malaysia Terengganu, Kuala Nerus 21030, Terengganu, Malaysia; karhyan93@gmail.com (T.K.Y.); asnu@umt.edu.my (A.A.); 2 Department of Agriculture, Quarantine and Crop Protection Section, Kuala Terengganu 20050, Terengganu, Malaysia; ppnt@terengganu.gov.my; 3 Institute of Tropical Biodiversity and Sustainable Development, Universiti Malaysia Terengganu, Kuala Nerus 21030, Terengganu, Malaysia

**Keywords:** red palm weevil (RPW), antifeedant activity, derivatives, eugenol, thymol

## Abstract

**Simple Summary:**

*Rhynchophorus ferrugineus* or the red palm weevil (RPW) is a major invasive insect pest that causes severe problems for palm cultivations and, in turn, for the palm industries worldwide. The current practice for controlling RPW in Malaysia mainly involves the use of synthetic pesticides. However, synthetic pesticides should not be encouraged because they are highly toxic and persistent in the environment and as such, can cause deterioration to the ecosystem and human health. Essential oil (EO) derivatives from eugenol and thymol were tested as potential natural compounds for controlling RPW. We evaluated the effect of eight EO derivatives from eugenol and thymol against the fourth instar larvae of RPW. Feeding contact bioassay was conducted for 14 days with different concentrations of these derivatives, in three replications for each treatment. All the derivatives showed feeding deterrent effects against RPW larvae, particularly 4-allyl-2-methoxy-1-(4-trifluoromethyl-benzyloxy)-benzene and 2-isopropyl-4-methyl-2-((4-nitrobenzyl) oxy) benzene, which demonstrated the most effective antifeedant properties in the eugenol and thymol derivative group. Overall, our findings revealed that both eugenol and thymol derivatives showed similar antifeedant activity against RPW larvae. However, the ether derivatives showed a stronger antifeedant effect than that of esters. Both eugenol and thymol are effective against RPW and have the potential to be investigated further as botanical insecticides for the management of RPW in the future.

**Abstract:**

Coconut palms in Malaysia are infested by a destructive invasive pest, RPW since 2007, and the pest’s population is difficult to control. At present, RPW control management mainly relies on the use of monocrotophos, which is administered by the trunk injection method. However, this pesticide can negatively impact human health and the ecosystem. Plant EO that can be used as a bio-pesticide is highly recommended as an alternative to monocrotophos because of its target-specific and eco-friendly properties. The antifeedant activity of eight eugenol and thymol derivatives from clove and thyme EOs were tested against the fourth instar larvae of RPW through oral bioassay for 14 days. Relative growth rate (RGR), relative consumption rate (RCR), the efficiency of conversion of ingested food (ECI), and the feeding deterrent index (FDI) were compared and analyzed. All of the derivatives showed antifeedant activity, particularly the eugenol derivative, 4-allyl-2-methoxy-1-(4-trifluoromethyl-benzyloxy)-benzene (FDI = 54.14%) and the thymol derivative, 2-isopropyl-4-methyl-2-((4-nitrobenzyl) oxy) benzene (FDI = 53.88%). Both of them showed promising results on their ability to be the most effective antifeedant agents in each derivative group. There was no significant difference in the effectiveness of the eugenol-based and thymol-based derivatives, but the ether derivative group (FDI = 45.63%) had a significantly stronger effect than the ester derivative group (FDI = 39.71%). This study revealed that the compound in ether form is more effective than the compound in ester form as an antifeedant agent against RPW larvae, regardless of the plant EO that the compound is derived from.

## 1. Introduction

Red palm weevil (RPW), *Rhynchophorus ferrugineus* Olivier (Coleoptera: Dryophthoridae), is an insect pest that causes damage to a broad spectrum of palm species around the world. This invasive species intruded into Malaysia in 2007 owing to the import of illegal date palm trees from the Middle East [1]. An intensive survey conducted across Terengganu state in 2011 revealed that RPW populations had increased drastically in all the eight districts of the state [2]. RPW then spread rapidly to Malaysia’s other states such as Perlis, Kedah, Pulau Pinang, and Kelantan in 2016 [3].

To date, RPW has been reported in an oil palm smallholding in Tumpat, Kelantan, and there was evidence of the oil palms there being affected [3]. At present, the estimated area of land under oil palm and coconut cultivations in Malaysia is about 6 million hectares [4,5]. Such wide planting area could provide favorable conditions for RPW to perpetuate their populations, thus possibly invading large areas of the plantations and causing drastic economic losses in Malaysia [6]. Several control strategies have been developed and used in the management of this pest, including the use of insecticides, cultural and sanitary control measures, pheromone traps, and biological control agents [7,8,9]. However, these RPW management methods are largely based on insecticide applications. Chemical control such as trunk injection using monocrotophos or methamidophos and the ground spraying technique using cypermethrin have also been used as the current standard operating procedure in Malaysia, but these synthetic pesticides have often shown properties of high toxicity and persistence in the environment [10]. Leaking of these pesticides into water sources could deteriorate other ecosystems and thus could harm living beings [11,12].

Currently, an integrated pest management (IPM) approach based on biological control rather than insecticides is being strongly promoted globally by policy-makers and international development agencies. In this context, many plant-based natural products are being tested, including EOs. EOs are reputed natural products utilized in many industries such as the food, cosmetics manufacturing, and pharmaceutical industries and in the production of botanical pesticides for use in the IPM approach. EOs perform a variety of functions such as those of a repellent, antifeedant, and attractor and shield from natural predators of herbivores [13,14].

Investigations on the efficacy of some plant EOs in controlling the RPW have focused mostly on the mortality percentage and antifeedant activity. For example, a study by Shukla et al. [15] showed that Crofton weed flower oil exhibited antifeedant activity against adult RPW. Ali et al. [16] found that orange and lemon oils as well as eucalyptus, basil, and castor oils can be used to control this pest at the egg and larval stages. Mohamed et al. [17] reported that nano-formulated EO of purslane showed good results against RPW larvae and adults similar to those shown by nano-formulated EOs of mustard and castor.

However, to date, the information available about the application of clove and thyme oil against RPW larvae is limited. It is believed that these plants, other than the family Arecaceae, possess certain chemical compounds that are effective in countering RPWs. Thus, this study tested the eugenol and thymol derivatives that were extracted from clove, i.e., *Syzygium aromaticum* Linnaeus (Myrtaceae) and thyme, i.e., *Thymus vulgaris* Linnaeus (Lamiaceae) were tested for their antifeedant activity against RPW larvae.

## 2. Materials and Methods

### 2.1. Pheromone Mass Trapping

Pheromone traps were used to collect RPW adults around coconut plantations in Kuala Nerus, Terengganu (5°22′17.6″ N 103°04′52.5″ E). The method adopted for RPW pheromone trapping was based on the method used by Wahizatul et al. [18] and Yong et al. [6] with slight modifications. A five-liter polypropylene bucket, with four holes (each with 3 cm diameter and 90° of angle apart from each other) on its upper part, was used. A small hole was poked on the lid for a nylon rope to hang the pheromone lure (700 mg synthetic ferrugineol sachet—Ferrolure P028+, ChemTica Int., Costa Rica). Pineapple slices (450 g) and 600 mL of water were placed into the bucket to enhance the trap’s effectiveness. The food baits were changed every fortnight, while sufficient water level was maintained to retain the required moisture in the trap. These pheromone traps were tied with raffia strings under the coconut trees to prevent them from being overturned by strong winds and being over-exposed to direct sunlight, which may change the conditions in the trap. The trapped RPWs were collected once a week and transferred to the laboratory for the rearing process.

### 2.2. Laboratory Rearing of RPW

Rearing of RPW adults and larvae was carried out according to the methods described by Zulklifi et al. [19]. The collected RPWs were reared in ventilated plastic containers (10 cm diameter × 5 cm height), under the laboratory conditions (25 ± 2 °C, 70 ± 5% RH, LD 12:12 photoperiod). Sugarcane slices were provided to adult RPWs as a food source and as the egg-laying substrate. Sugarcane slices were replaced once every four days, and the fiber part of used sugarcanes was ripped apart to check the presence of eggs or larvae. The eggs were then transferred to sago palm stems, which were used as substrate and were cut and bored with a small hole to place the eggs. The eggs were then examined until they had hatched within 5 days, while each neonate larva was transferred to a small, ventilated plastic container (3.6 cm diameter × 3.2 cm height) that contained a sago palm stem as food. The sago palm stem was renewed once every week unless it was completely consumed earlier by the larvae. The width of the head capsules of the larvae was measured using a digital vernier caliper to determine the instars according to Dyar’s ratio [20]. Only healthy fourth-instar larvae were selected because larger larval size allowed easier handling during the experiments. Neonates and early instar larvae were avoided as they were too small, difficult to be detected in the food substrate, and they could easily be injured by the forceps used during the food replacement. The amount of food consumption in the fourth instar larvae was also significantly different from the third and fifth instars, which helped provide a more accurate and consistent measurement during the experiments [19].

### 2.3. Source of Eugenol and Thymol Derivatives

Eight eugenol and thymol derivatives were obtained from Nurul Hazwani, C.A.R., and Asnuzilawati, A., Universiti Malaysia Terengganu [21,22]. All the derivatives used were 100% pure. There were four derivatives (two ethers and two esters) in both eugenol and thymol groups (Table 1).

### 2.4. Bioassay

A series of dilutions for the eight derivatives of eugenol and thymol were prepared using acetone as the solvent to obtain concentrations of 200 ppm, 400 ppm, and 600 ppm. The control was treated with an acetone solvent (99.79%, HmbG Chemicals Progressive Scientific Pvt. Ltd., Selangor, Malaysia) only. Each treatment was replicated thrice, and each treatment replication consisted of three larvae. A total of 225 larvae were used in this study.

Sago stem was selected as the food substrate as it is locally available and cheap. Sago is also one of the host plants for RPW [23]. Each pre-weighed sago palm stem was cut into a block of 3 cm × 2 cm × 1.5 cm. It was then soaked in 2 mL of acetone or derivatives in certain concentrations in a petri dish for 1 min. The solvent was allowed to evaporate at room temperature for about an hour. A hole was bored for the larvae to grub and feed inside the sago palm stem. Each pre-weighed larva was starved for three hours before it was used in the bioassay experiment. Each food and larva unit was then transferred to a ventilated plastic container (6 cm diameter × 3.9 cm height). The food was replaced daily, and the weight of food consumed and that of the larvae were measured and recorded daily for 14 days using an electronic balance of 0.01 g (RADWAG WTB 2000).

Relative growth rate (RGR), relative consumption rate (RCR), efficiency of conversion of ingested food (ECI), and feeding deterrent index (FDI) were calculated according to the following equations [14]:

RGR = (A − B)/(B × day) A = Weight of the larva after the experiment B = Weight of the larva before the experiment(1)

RCR = D/(B × day) D = Weight of the food consumed by the larva(2)

ECI = RGR/RCR × 100%(3)

FDI = [(C − T)/C] × 100% C = Weight of the food consumed in control T = Weight of the food consumed in treatment(4)

### 2.5. Data Analysis

All data were statistically analyzed using the mean values for the three replications carried out in each treatment. The results were taken only if the larva survived at least for one week. This was done in order to obtain the most reliable data. One-way ANOVA followed by Tukey’s HSD post hoc test was used to determine significant differences in the antifeedant activities among eugenol derivatives, thymol derivatives, and the control. Further, a T-test was applied to compare the efficacy between eugenol-based and thymol-based groups and the different functional groups (ester and ether) therein. All the significance difference levels were determined at α = 0.05. All statistical analyses were conducted using SPSS (version 21.0).

## 3. Results

### 3.1. Eugenol Derivatives

The range of daily consumption amounts in the treatments ranged from 0.17 g/day to 0.25 g/day. Generally, the daily consumption amount in all the treatments was significantly lower by at least 35% than the daily consumption amount in control (ANOVA, df = 38; F = 60.759, *p* < 0.001). The lowest food consumption amount among all treatments was observed in 4-allyl-2-methoxy-1-(4-trifluoromethyl-benzyloxy)-benzene (WN16) with 400 ppm. This value was significantly different from the other derivatives with the same concentration (ANOVA, df = 11; F = 33.536, *p* < 0.001) (Table 2).

RGR of the larvae was analyzed after 14 days. Negative RGR values indicated that the weight of the larvae decreased after the treatment. Overall, there was no significant difference between the RGR values of all the treatments and the control or in each treatment with different concentrations (ANOVA, df = 38; F = 1.55, *p* = 0.169).

RCR refers to the feeding rate (minimum = 0; maximum = 1.0) of the larvae during the experimental periods. Most of the larvae that fed on treated food in all the concentrations exhibited lower RCR values than the control within the 14-day duration of the experiment. However, the larvae that fed on 4-allyl-2methoxyphenyl 4-ethylbenzoate (WN14) in concentrations of 400 ppm and 600 ppm had no significant difference compared with the control. ECI was calculated by taking into account the data of RGR and RCR arrived at by the aforementioned methods.

The ECI value indicates the rate of the ingested food that gets converted into larval growth rate. A higher ECI value implies that more of the consumed food was ingested and converted into a supplement for growing. There was no significant difference between ECIs of the control and all the treatments with different concentrations (ANOVA, df = 38; F = 2.028, *p* = 0.064). It was found that 4-allyl-2-methoxyphenyl 4-bromobenzoate (WN15) had a significantly higher ECI value in 200 ppm compared with the ECI value of 4-allyl-2-methoxy-1-(4-nitrobenzyloxy)-benzene (WN11) in 400 ppm.

FDI shows the effectiveness of the antifeedant activity in all the treatments. The higher the FDI value, the stronger was the antifeedant effect and, hence, the lesser the food consumed by the larvae. All the treatments showed a feeding deterrent effect, and the FDI value ranged between 36% and 55%. On the other hand, in 200 ppm, the compound with the highest FDI value was found to be WN16, but it showed no significant difference with WN11. In 400 ppm, WN16 showed the highest FDI value, which was significantly higher than the values of all the other compounds in the same concentration. The FDI values of all the compounds in 600 ppm showed no significant difference with each other.

All the compounds were then classified into their functional groups, namely ether for WN11 and WN16 and ester for WN14 and WN15. The mean values of FDI for each of the groups were calculated and compared with each other. It was found that the mean FDI value for ethers was significantly higher than the mean FDI value for esters (T-test, df = 34, 2-tailed; α < 0.05) (Table 3).

The effectiveness sequence was arranged by the following steps: a comparison through RGR, RCR, ECI, and FDI; a comparison of FDI among the derivatives (in the same concentration); and a comparison of FDI among concentrations in the same derivative. The results from RGR and ECI were not suitable to compare the effects of these compounds given that the treatments had no significant difference when it came to the control. Further, no significant differences in RCR were detected in the control and WN14 (in 400 ppm and 600 ppm), which indicates that WN14 had the lowest antifeedant activity among the eugenol derivatives. WN16 expressed the highest FDI value (54.14%), and it was the most effective eugenol derivative. The FDI values of WN11 and WN15 were similar, but the FDI value of WN15 in 400 ppm had no significant difference from that of WN14 in 400 ppm. Thus, in terms of effectiveness, these four compounds can be ranked as follows: WN16 > WN11 > WN15 > WN14.

### 3.2. Thymol Derivatives

The daily consumption rates in all the treatments were significantly lower by at least 35% than control. Among them, 2-isopropyl-4-methyl-2-((4-nitrobenzyl) oxy) benzene (WNT2) in 200 ppm presented the lowest food consumption rate (0.178 ± 0.003 g/day) (ANOVA, df = 11; F = 15.213, *p* = 0.001), followed by WNT2 in 400 ppm, which was also significantly different from other compounds with the same concentration (ANOVA, df = 38; F = 116.34, *p* < 0.001) (Table 4).

RGR’s range for treatments was between −0.001 g/g day and 0.005 g/g day. There was no significant difference between the RGR values of each treatment with different concentrations and control (ANOVA, df = 38; F = 0.935, *p* = 0.529). The differences in RCR between the control and treated larvae were up to 0.24 g/g day. All the larvae that fed on treated food in all the concentrations showed a lower RCR value than the control. There was no significant difference between the ECI for control and each of the treatment groups with different concentrations (ANOVA, df = 38; F = 1.478, *p* = 0.195).

All the treatments exhibited a feeding deterrent effect, and the FDI values ranged from 37% to 54%. When compared with the findings in 200 ppm, it was found that the compound with the highest FDI value was WNT2, which was also significantly different from the values of the other compounds in the same concentration. In 400 ppm too, WNT2 had the highest FDI value, and it was significantly higher than the values of all the other compounds in the same concentration. In 600 ppm, 2-(benzyloxy)-1-isopropyl-4-methylbenzene (WNT1) had the highest FDI values, but it showed a significant difference only in the case of 2-isopropyl-5-methylphenyl 4-ethylbenzoate (WNT5).

All thymol derivatives were classified into their respective functional groups, which were ether for WNT1 and WNT2 and ester for 2-isopropyl-5-methylphenyl 4-bromobenzoate (WNT4) and WNT5. The mean value of FDI within these groups was calculated and compared (Table 5). The mean FDI value for ethers was significantly higher than the mean FDI value of esters (T-test, df = 34, 2-tailed; α < 0.05).

The antifeedant activities of the four thymol derivatives were evaluated based on the criteria mentioned in Section 3.1. No statistical significance was found among the results of the treatments in RGR, RCR, and ECI. So, they are not sufficient as a comparison reference on the effectiveness of these compounds. Based on the FDI values, WNT2 was the most effective derivative and was able to deter the feeding rate by up to 50% in a lower concentration. Further, based on the results, the FDI values of WNT1, WNT4, and WNT5 (in 200 ppm and 400 ppm) were not significantly different from each other. However, the FDI value of WNT1 in 600 ppm was the highest, while that of WNT5 in the same concentration was found to be the lowest. Hence, the effectiveness sequence of the thymol derivatives can be listed as WNT2 > WNT1 > WNT4 > WNT5.

### 3.3. Comparison of Eugenol and Thymol Zerivatives

All the eight derivatives in each base were grouped and compared with each other as presented in Table 6 (eugenol-based–WN11, WN14, WN15, and WN16; thymol-based–WNT1, WNT2, WNT4, and WNT5). The thymol-based derivatives had a higher FDI value than the eugenol-based derivatives, but there was no significant difference between these two values (T-test, df = 70, 2-tailed; α = 0.413). Thereafter, the eight derivatives in each functional group were grouped and compared as depicted in Table 7 (ether–WN11, WN16, WNT1, and WNT2; ester–WN14, WN15, WNT4, and WNT5). The mean of the FDI values for each group was also calculated, and the ether compounds showed a significantly higher FDI value than the ester compounds (T-test, df = 70, 2-tailed; α < 0.05).

The eight derivatives were arranged based on the effectiveness of their antifeedant activity, and their sequence got listed as WNT2 > WN16 > WN11 > WNT1 > WN15 > WNT4 > WNT5 > WN14. WNT2 was listed as the most effective derivative as its FDI value was the highest in the 200 ppm concentration. WNT2 was followed by WN16, which had the highest FDI value in the 400 ppm concentration, but no significant difference was detected between WN16 and WNT2 in the same concentration.

## 4. Discussion

Larval feeding is crucial for its growth and optimal development according to its lifecycle. The quality and quantity of the food administered were important aspects for their development. Therefore, this study focused on regulating or deterring the feeding of the RPW larvae by continually providing food added with the necessary compounds, without any lethal effect (acute toxicity).

The daily food consumption rate of the control, which was treated with acetone, was compared with the one found by Zulkifli et al. [19]. They had reported that the daily food consumption rate of a third instar larva on a sago palm stem was 0.31 ± 0.01 g/day and the mean amount of a sago stem consumed by a larva until its pupa stage was 0.4 g/day. In this study, the consumption rate of the fourth instar in control was slightly higher, but it was close to the mean value. These findings supported that the control value was in an acceptable range. The rate of consumption for eugenol or thymol in the current study was lower compared with that found by Zulkifli et al. [19]. For instance, the first instar larvae in the said study consumed 0.24 ± 0.01 g/day of the sago stem that they were fed [19], whereas, in this study, the third instar larvae consumed 0.17 + 0.01 g/day (applying WNT2). This proves that eugenol and thymol have deterred the feeding behavior of the RPW larvae.

According to Price and Berry [24], eugenol mimics octopamine and may induce cellular changes in cloned cells in the nervous system of insects, which accounts for its insecticidal properties. They had demonstrated that eugenol depressed spontaneous and stimulus-evoked impulses in the abdominal nerve cords of cockroaches with an almost complete block of spikes. Kim & Lee [25] have reported that basil oil and its components could be potential candidates as fumigants for the control of adult grain storage insect pests, *Sitophilus zeamais* (Motsch.) (Coleoptera: Curculionidae) and *Tribolium castaneum* (Herbst) (Coleoptera: Tenebrionidae). The repellent action of the tested oils, which was introduced through vapors, affected the targeted pests’ respiratory systems or interacted with their receptors, thereby blocking their sense of smell.

The most effective antifeedant agent, WN16 (4-allyl-2-methoxy-1-(4-trifluoromethyl-benzyloxy)-benzene) may have contributed to the tri-fluorinated structure in this compound [22]. The compounds which comprised tri-fluorinate structures in previous studies had similar effects on insect behavior. Renou et al. [26] have reported that 3-Octylthio-1,1,1-trifluoropropan-2-one (OTFP) acts as an anti-esterase agent to counter insect behavior, while Bau et al. [27] have shown that trifluoromethyl ketone (TFMK) could disrupt the flight behaviors of the Egyptian armyworm, *Spodoptera littoralis* (Boisd.) (Lepidoptera: Noctuidae) and the Mediterranean corn borer, *Sesamia nonagriodes* (Lefebvre) (Lepidoptera: Noctuidae), through the olfactory system by their antennae or setae. Hence, a tri-fluorinated compound may adversely affect the feeding activity of RPW larvae.

The effect of thymol derivatives has been studied earlier for its insecticidal activity against many types of insects such as *Culex pipiens* (Linnaeus) (Diptera: Culicidae) [28], *Aedes aegypti* (Linnaeus) (Diptera: Culicidae) [29], and *Sitophilus granarius* (Linnaeus) (Coleoptera) [30]. Thymol has also shown to have an adverse effect on the walking behavior of *Sitophilus zeamais* [31]. However, to date, the number of studies conducted on the efficacy of thymol as a promising biopesticide for the control of RPW has been limited.

WNT2 (2-isopropyl-4-methyl-2-((4-nitrobenzyl) oxy) benzene) comprises nitrogen dioxide (NO_2_) in its molecular structure. The continuous contact of NO_2_ results in allosteric inhibition of Glutathione S-Transferase (GST) enzyme, which can produce a detoxification function in larval bodies [32,33]. Based on the aforementioned studies, the accumulation of WNT2 could temporarily or permanently inhibit the RPW larval detoxification pathway when the allosteric site of etherase is occupied by NO_2_. In addition, WN11 also has a composition of NO_2_ in eugenol derivatives like WNT2, while WN11 was placed second only to WN16 in the effectiveness of the antifeedant activity. This indicates that the NO_2_ structure may cause a negative impact toward the RPW larvae and in determining the effectiveness ranking of the derivatives. The results implied that eugenol and thymol derivatives were not affecting the growth mechanisms of the RPW larvae, including food digestion and growth hormone inhibition. However, there was a decline in RPW larvae’s food intake, which could possibly be the outcome of these compounds entering them through other sensory systems such as the olfactory and gustatory systems [34]. As an example, Jacobson et al. [35] have reported that ether extract of neem was effective in repelling dried-fruit beetles, *Carpophilus hemipterus* (Linnaeus) (Coleoptera: Nitidulidae), in both adult and larval stages, whereas it also proved that the extract’s antifeedant compound could carry out its function without affecting its digestive system.

The antifeedant activity of the eugenol derivatives did not significantly differ from the thymol derivatives. Isman et al. [36] have reported that EOs of clove and thyme often act as insecticides which affect the octopamine receptors of insects, but these EOs do not harm those mammals that lack this receptor. Hosozawa et al. [37] have examined 13 antifeedant compounds from plants against *Spodoptera litura* (Lepidoptera: Noctuidae), and they found that some compounds shared the same antifeedant activity. Eugenol and thymol were also tested on green peach aphid, and both these compounds showed not only behavioral effects but also toxic effects on the insect. From a functional perspective, both eugenol and thymol share the immobilization function of antimicrobials [38]. Both these EOs also worked as anesthetics (causing short-term paralysis) in common carp [39]. From the above cases, eugenol and thymol can be suggested to share the same functions to kill not only insects but also other organisms.

These derivatives were also classified into a group according to their functional grouping, which involved ether and ester. It was interesting to find that ethers were significantly stronger antifeedant agents than esters. From the effectiveness sequence above, four derivatives (WN11, WN16, WNT1, and WNT2) that showed stronger antifeedant activity consisted of ether functional groups, while four weaker derivatives (WN14, WN15, WNT4, and WNT5) were esters. The stability of a compound is one of the factors that can alter the effectiveness of its antifeedant activity. All ethers analyzed in this study were found to have more stable characteristics than all the esters analyzed in this study [40], and they were found to be difficult when it came to reacting with both oxidizing and reducing agents. Esters, which lack hydrogen, bonded with the oxygen molecules and caused them to end up with a lower bonding strength and ability to break down easily in the presence of water. The presence of water and enzymes in the larval body breaks down these esters into ethanoic acid and ethanol through hydrolysis. Since water is the most essential element for the growth and detoxification of living organisms, esters could easily degrade within their body, whereas ethers required alcohol to break down [40].

The detoxification action in RPW larvae may also explain why ethers are stronger antifeedant agents than esters. According to Wang et al. [41], RPW fat accumulation depends on the larval stage, and the enrichment of lipid metabolism is crucial for the insect pest’s entire life cycle. The holometabolous insects occupy most of their time feeding in the larval stage and storing nutrients and fat, while the composition of nonspecific esterase is highly distributed in the insect fat tissue. Esterases play a very important role in dietary detoxification and developing insecticide resistance. This is because of the nonspecific substrate affinity of these enzymes toward many different compounds [42,43]. Thus, the presence of certain nonspecific esterases in the RPW larval body may probably be breaking down the ester compounds to a low level of toxic material. The most common example of etherase is glutathione S-transferase (GST), which is an important cellular component of detoxification. GSTs are usually associated with glutathione, which alters the conjugation of states to carry out the redox reaction. In all, six types of GSTs have been reported in insects, but most of these GSTs’ detoxification effects remain unclear as they can synergize and fuse to break down different xenobiotic substances. Furthermore, GSTs are classified as etherase, but they have broad binding specificity, and some of them have a substrate preference for esterase activity. GSTs’ compositions in the larvae of a similar genus, the African palm weevil, *Rhynchophorus phoenicis* (Fabricius) (Coleoptera: Dryophthoridae) were evaluated, and showed that glutathione peroxides (GPX), glutathione reductase (GR), and GST were found in them [44].

Further experiments on these compounds need to be conducted on the other stages of RPW’s life cycle, including the adult and egg stages. It is hoped that the findings of his study will contribute toward exploring the potential of eugenol and thymol as botanical pesticides for developing a sustainable agricultural pest management system for RPW.

## 5. Conclusions

The findings of this study have revealed the antifeedant activity of eugenol and thymol derivatives against the RPW larvae, with 4-allyl-2-methoxy-1-(4-trifluoromethyl-benzyloxy)-benzene and 2-isopropyl-4-methyl-2-((4-nitrobenzyl) oxy) benzene being indicated as the most effective compounds from the two groups respectively. Furthermore, a similar effect was found in the case of eugenol and thymol derivatives, where the ethers have significantly stronger effects on the RPW larvae than esters. Therefore, both these compounds have the potential to be used as targeted bioinsecticides for the management of RPW.

## Figures and Tables

**Table 1:** Information regarding eugenol and thymol derivatives.

Series	Code	Name	Class	Molecular Structure	Appearance
Eugenol derivative	WN11	4-allyl-2-methoxy-1-(4-nitrobenzyloxy)-benzene	Ether	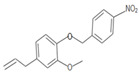	Yellow solid
	WN16	4-allyl-2-methoxy-1-(4-trifluoromethyl-benzyloxy)-benzene	Ether	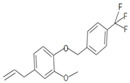	White solid
	WN14	4-allyl-2methoxyphenyl 4-ethylbenzoate	Ester	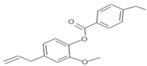	White solid
	WN15	4-allyl-2-methoxyphenyl 4-bromobenzoate	Ester	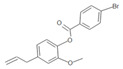	White solid
Thymol derivative	WNT1	2-(benzyloxy)-1-isopropyl-4-methylbenzene	Ether	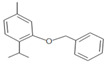	Oily solution
	WNT2	2-isopropyl-4-methyl-2-((4-nitrobenzyl) oxy) benzene	Ether	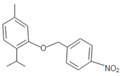	Yellowish orange solid
	WNT4	2-isopropyl-5-methylphenyl 4-bromobenzoate	Ester	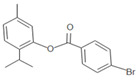	Oily yellow solution
	WNT5	2-isopropyl-5-methylphenyl 4-ethylbenzoate	Ester	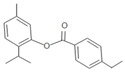	Oily yellow solution

**Table 2:** Mean ± SE of data for RPW larvae that were treated with eugenol derivatives.

Treatment	Concentration (ppm)	Consumption (g/day)	RGR (g/g day)	RCR (g/g day)	ECI	FDI (%)
Control	0	0.386 ± 0.013 *	−0.011 ± 0.01	0.316 ± 0.039 a	−9.156 ±1.8 ab	-
WN11	200	0.215 ± 0.003 a,A	−0.008 ± 0.00	0.090 ± 0.005 b	−2.120 ± 4.38 ab	44.22 ab,A
	400	0.207 ± 0.002 b,A	−0.016 ± 0.01	0.095 ± 0.007 b	−22.78 ± 7.98 a	46.37 b,A
	600	0.228 ± 0.0004 a,B	−0.027 ± 0.02	0.159 ± 0.041 b	−14.93 ± 12.9 ab	40.73 a,A
WN14	200	0.240 ± 0.001 a,AB	−0.001 ± 0.01	0.1692 ± 0.024 b	2.899 ± 8.21 ab	37.74 a,A
	400	0.241 ± 0.002 d,B	−0.006 ± 0.02	0.214 ± 0.029 ab	0.733 ± 18.15 ab	37.43 a,A
	600	0.235 ± 0.0006 ab,A	0.019 ±0.01	0.212 ± 0.058 ab	19.15 ± 11.18 ab	39.13 a,A
WN15	200	0.237 ± 0.002 a,C	0.017 ± 0.001	0.096 ± 0.003 b	25.72 ± 1.51 b	38.47 ab,A
	400	0.218 ± 0.001 c,A	−0.023 ± 0.01	0.129 ± 0.014 b	−7.499 ± 7.25 ab	43.49 ab,A
	600	0.225 ± 0.0008 a,B	−0.007 ± 0.01	0.112 ± 0.013 b	2.10 ± 11.04 ab	41.71 a,A
WN16	200	0.210 ± 0.014 a,AB	−0.005 ± 0.01	0.099 ± 0.013 b	−3.127 ± 7.51 ab	45.74 b,B
	400	0.177 ± 0.004 a,A	−0.009 ± 0.02	0.093 ± 0.021 b	−8.855 ± 12.0 ab	54.14 c,C
	600	0.245 ± 0.006 b,B	−0.022 ± 0.00	0.143 ± 0.023 b	−15.16 ± 2.27 ab	36.59 a,A

The symbol * indicates that it is significantly higher when compared with all other treatments (*p* < 0.05). The same small letter indicates that there was no significant difference between the derivatives at the same concentration (*p* > 0.05). The same capital letter indicates that no significant difference existed between concentrations in each derivative (*p* > 0.05).

**Table 3:** Group statistics (FDI) (%) between functional groups of eugenol derivatives.

Functional Group	N	Mean	Standard Error
Ether	18	44.63 *	1.40
Ester	18	39.66	0.81

The symbol * indicates their higher value and significant difference with each other (α < 0.05). N = total number of treated larvae from ether and ester groups in eugenol derivatives.

**Table 4:** Mean ± SE of data for RPW larvae that were treated with thymol derivatives.

Treatment	Concentration (ppm)	Consumption (g/day)	RGR (g/g day)	RCR (g/g day)	ECI	FDI (%)
Control	0	0.386 ± 0.013 *	−0.011 ± 0.01	0.316 ± 0.039 a	−9.156 ±1.8 ab	-
WNT1	200	0.213 ± 0.001 b,B	−0.002 ± 0.01	0.075 ± 0.010 b	−5.213 ± 7.496	44.72 a,A
	400	0.221 ± 0.001 b,C	−0.012 ± 0.01	0.089 ± 0.005 b	−12.146 ± 8.568	42.64 a,A
	600	0.206 ± 0.0006 a,A	−0.027 ± 0.01	0.101 ± 0.015 b	−27.446 ± 4.889	46.52 b,A
WNT2	200	0.178 ± 0.003 a,A	−0.005 ± 0.00	0.096 ± 0.015 b	0.489 ± 5.774	53.88 b,B
	400	0.189 ± 0.004 a,B	−0.011 ± 0.02	0.121 ± 0.043 b	1.459 ± 14.451	50.91 b,B
	600	0.227 ± 0.001 b,C	0.001 ± 0.02	0.116 ± 0.007 b	3.523 ± 16.979	41.14 ab,A
WNT4	200	0.222 ± 0.002 c,A	−0.024 ± 0.01	0.109 ± 0.024 b	−21.626 ± 1.375	42.49 a,A
	400	0.240 ± 0.002 c,B	−0.009 ± 0.00	0.081 ± 0.009 b	−11.349 ± 3.949	37.64 a,A
	600	0.227 ± 0.0005 b,A	−0.009 ± 0.00	0.073 ± 0.004 b	−11.906 ± 1.095	41.10 ab,A
WNT5	200	0.229 ± 0.001 c,A	0.005 ± 0.00	0.092 ± 0.003 b	0.175 ± 2.723	40.62 a,A
	400	0.237 ± 0.003 c,AB	−0.014 ± 0.01	0.102 ± 0.015 b	−11.607 ± 2.622	38.58 a,A
	600	0.238 ± 0.0007 c,B	−0.003 ± 0.01	0.110 ± 0.008 b	−3.595 ± 4.371	38.13 a,A

The symbol * indicates that it is significantly higher in all the other treatments (*p* < 0.05). The same small letter indicates that no significant difference exists between the derivatives at the same concentration levels (*p* > 0.05). The same capital letter indicates that there were no significant differences between concentrations in each derivative (*p* > 0.05).

**Table 5:** Group statistics (FDI) (%) compared among functional groups of thymol derivatives.

Functional Group	N	Mean	Standard Error
Ether	18	46.64 *	1.40
Ester	18	39.76	0.81

The symbol * indicates a higher value and significant difference with each other (α < 0.05). N = total number of treated larvae from ether and ester groups in thymol derivatives.

**Table 6:** Group statistics (FDI) (%) between eugenol and thymol derivatives.

Functional Group	N	Mean	Standard Error
Eugenol	36	42.15	0.8993
Thymol	36	43.20	0.9046

No significant difference was found in the comparison of eugenol and thymol derivatives. N = total number of treated larvae from eugenol and thymol derivatives.

**Table 7:** Group statistics (FDI) (%) between functional groups of thymol derivative.

Functional Group	N	Mean	Standard Error
Ether	36	45.63 *	0.92
Ester	36	39.71	0.54

The symbol * indicates the higher value and significant difference with each other (α < 0.05). N = total number of treated larvae from two types of ether/ester in thymol derivative.

## Data Availability

Not applicable.
